# Effect of phonons on the ac conductance of molecular junctions

**DOI:** 10.1186/1556-276X-6-204

**Published:** 2011-03-09

**Authors:** Akiko Ueda, Ora Entin-Wohlman, Amnon Aharony

**Affiliations:** 1 Department of Physics, Ben Gurion University, Beer Sheva 84105, Israel; 2 Tel Aviv University, Tel Aviv 69978, Israel

## Abstract

We theoretically examine the effect of a single phonon mode on the structure of the frequency dependence of the ac conductance of molecular junctions, in the linear response regime. The conductance is enhanced (suppressed) by the electron-phonon interaction when the chemical potential is below (above) the energy of the electronic state on the molecule.

PACS numbers: 71.38.-k, 73.21.La, 73.23.-b

## Introduction

Molecular junctions, made of a single molecule (or a few molecules) attached to metal electrodes, seem rather well established experimentally. An interesting property that one can investigate in such systems is the interplay between the electrical and the vibrational degrees of freedom as is manifested in the *I*-*V *characteristics [1,2].

To a certain extent, this system can be modeled by a quantum dot with a single effective level *ε*_0_, connected to two leads. When electrons pass through the quantum dot, they are coupled to a single phonon mode of frequency *ω*_0_. The dc conductance of the system has been investigated theoretically before, leading to some distinct hallmarks of the electron- phonon (e-ph) interaction [3-6]. For example, the Breit-Wigner resonance of the dc linear conductance (as a function of the chemical potential *μ*, and at very low temperatures) is *narrowed down *by the e-ph interaction due to the renormalization of the tunnel coupling between the dot and the leads (the Frank-Condon blockade) [4,5]. On the other hand, the e-ph interaction *does not *lead to subphonon peaks in the linear response conductance when plotted as a function of the chemical potential. In the nonlinear response regime, in particular for voltages exceeding the frequency *ω*_0 _of the vibrational mode, the opening of the inelastic channels gives rise to a sharp structure in the *I*-*V *characteristics. In this article, we consider the ac linear conductance to examine phonon-induced structures on transport properties when the ac field is present.

## Model and calculation method

We consider two reservoirs (L and R), connected via a single level quantum dot. The reservoirs have different chemical potentials, *μ*_L _= *μ*+Re[*δμ*_L_*e^iωt^*] and *μ*_R _= *μ*+Re[*δμ*_R_*e^iωt^*]. When electrons pass through the quantum dot, they are coupled to a single phonon mode of frequency *ω*_0_. In its simplest formulation, the Hamiltonian of the electron-phonon (e-ph) interaction can be written as , where *b *(*c*_0_) and *b*^† ^() are the annihilation and the creation operators of phonons (electrons in the dot), and *γ *is the coupling strength of the e-ph interaction. The broadening of the resonant level on the molecule is given by Γ = Γ_L _+ Γ_R_, with , where ν is the density of states of the electrons in the leads and *t*_L(R) _is the tunneling matrix element coupling the dot to the left (right) lead.

The ac conductance of the system is derived by the Kubo formula. In the linear response regime, the current is given by *I *= (*I*_L _*- I*_R_)/2, where(1)

Here,  is the Fourier transform of the two particle Green function,(2)

where , with  and *c*_*k*(*p*) _denoting the creation and annihilation operators of an electron of momentum *k*(*p*) in the left (right) lead. The ac conductance is then given by(3)

In this article we consider the case of the symmetric tunnel coupling, Γ_L _= Γ_R_. We also assume *δμ*_L _= - *δμ*_R _= *δμ*/2. The e-ph interaction is treated by the perturbation expansion, to order *γ*^2^. The resulting conductance includes the self-energies stemming from the Hartree and from the exchange terms of the e-ph interaction, while the vertex corrections of the e-ph interaction vanish when the tunnel coupling is symmetric. We also take into account the RPA type dressing of the phonon, resulting from its coupling with electrons in the leads [3].

## Results

**Figure 1:**
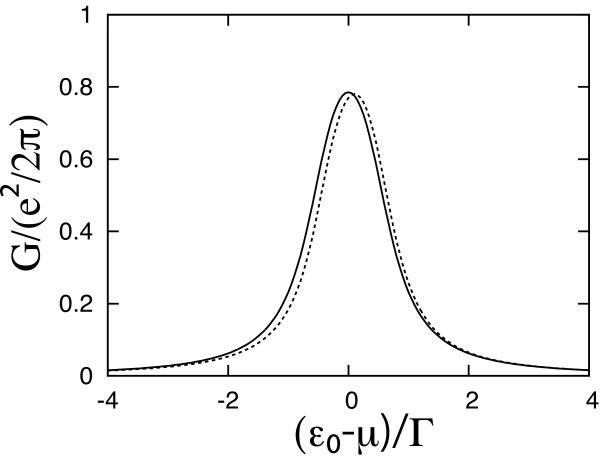
**The ac conductance as a function of (*ε*_0 _- *μ*)**. The ac frequency *ω *= Γ. Γ_L _= Γ_R _and *δμ*_L _= -*δμ*_R_. Solid line: without e-ph interaction. Dotted line: *γ *= 0.3Γ and *ω*_0 _= Γ.

The total conductance is given by *G *= *G*_0 _+ *G*_int_, where *G*_0 _is the ac conductance without the e-ph interaction, while *G*_int _≡ *G*_H _+ *G*_ex _contains the Hartree contribution *G*_H _and the exchange term *G*_ex_. Figure 1 shows the conductance *G *as a function of *ε*_0 _- *μ*, for a fixed ac frequency *ω *= 0.5Γ. The solid line indicates *G*_0_. The dotted line shows the full conductance *G*, with *γ *= 0.3Γ. The peak becomes somewhat narrower, and it is shifted to higher energy, which implies a lower (higher) conductance for *ε*_0 _* < μ *(*ε*_0 _* > μ*). However, no additional peak structure appears.

**Figure 2:**
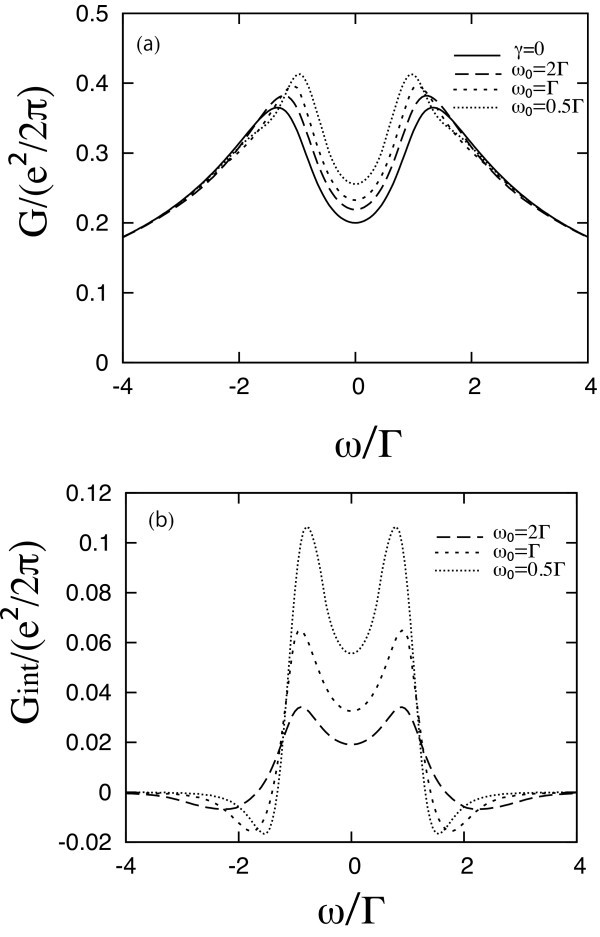
**The ac conductance as a function of the ac frequency *ω *at *ε*_0 _- *μ *= Γ**. **(a) **The total conductance when Γ_L _= Γ_R _and *δμ*_L _= -*δμ*_R_. The broken lines indicate the conductance in the presence of e-ph interaction with *γ *= 0.4Γ. *ω*_0 _= 2Γ, or 0.5Γ. The solid line is the 'bare' conductance *G*_0_, in the absence of e-ph interaction. **(b) **The additional conductance due to the e-ph interaction, *G*_int_(*ω*) = *G*_H_(*ω*) + *G*_ex_(*ω*), for the same parameters as in **(a)**.

**Figure 3:**
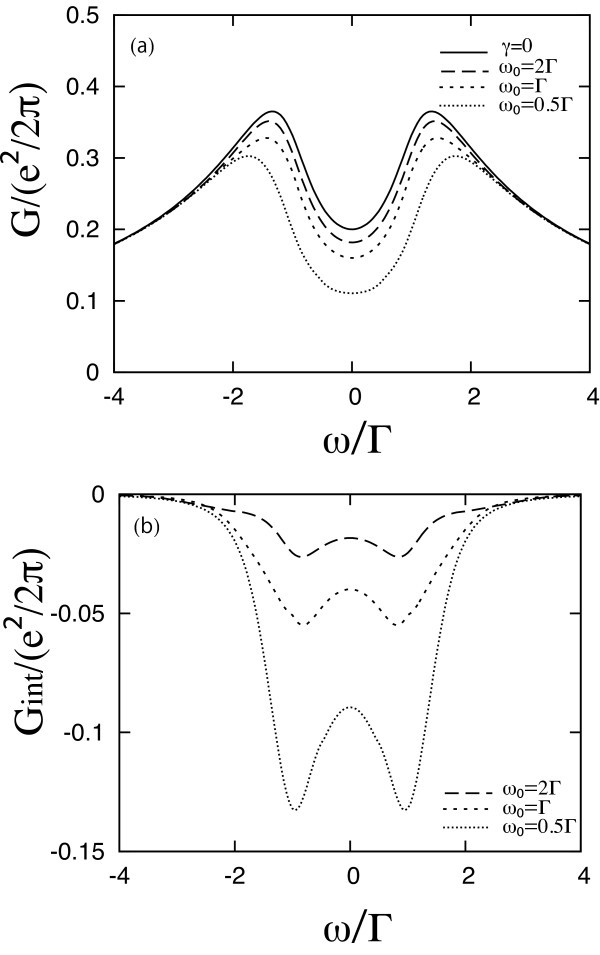
**The conductance as a function of the ac frequency *ω *at *ε*_0 _- *μ *= -Γ**. **(a) **The total conductance when Γ_L _= Γ_R _and *δμ*_L _= -*δμ*_R_. The broken lines indicate the conductance in the presence of e-ph interaction with *γ *= 0.3Γ. *ω*_0 _= 2Γ, Γ or 0.5Γ. The solid line is the 'bare' conductance *G*_0 _in the absence of e-ph interaction. **(b) **The additional conductance due to the e-ph interaction, *G*_int_(*ω*) = *G*_H_(*ω*) + *G*_ex_(*ω*), for the same parameters as in **(a)**.

Next, Figure 2a shows the full ac conductance *G *as a function of the ac frequency *ω*, when *ε*_0 _- *μ *= Γ. The solid line in Figure 2a indicates *G*_0_. Two broad peaks appear around *ω *of order ± 1.5(*ε*_0 _- *μ*). The broken lines show *G *in the presence of the e-ph interaction with *ω*_0 _= 2Γ, *ω*_0 _= Γ, or *ω*_0 _= 0.5Γ. The e-ph interaction increases the conductance in the region between the original peaks, shifting these peaks to lower *|ω|*, and decreases it slightly outside this region. Figure 2b indicates the additional conductance due to the e-ph interaction, *G*_int_, for the same parameters. Similar results arise for all positive *ε*_0 _- *μ*. Both *G*_H _and *G*_ex _show two sharp peaks around *ω *~ ± (*ε*_0 _- *μ*) (causing the increase in *G *and the shift in its peaks), and both decay rather fast outside this region. In addition, *G*_ex _also exhibits two negative minima, which generate small 'shoulders' in the total *G*. For *ε*_0 _*>μ*, *G*_int _is dominated by *G*_ex_. The exchange term virtually creates a polaron level in the molecule, which enhances the conductance. The amount of increase is more dominant for lower *ω*_0_. The situation reverses for *ε*_0 _*<μ*, as seen in Figure 3. Here, *G*_0 _remains as before, but the ac conductance is suppressed by the e-ph interaction. Now *G*_int _is always negative, and is dominated by *G*_H_. The Hartree term of the e-ph interaction shifts the energy level in the molecule to lower values, resulting in the suppression of *G*. The amount of decrease is larger for lower *ω*_0_.

## Conclusion

We have studied the additional effect of the e-ph interaction on the ac conductance of a localized level, representing a molecular junction. The e-ph interaction enhances or suppresses the conductance depending on whether *ε*_0 _* > μ *or *ε*_0 _*< μ*.

## Abbreviations

e-ph: Electron-phonon.

## Competing interests

The authors declare that they have no competing interests.

## Authors' contributions

AU carried out the analytical and numerical calculations of the results and drafted the manuscript. OE conceived of the study. AA participated in numerical calculations. All authors discussed the results and commented and approved the manuscript.
